# Development and validation of a novel 3-gene prognostic model for pancreatic adenocarcinoma based on ferroptosis-related genes

**DOI:** 10.1186/s12935-021-02431-8

**Published:** 2022-01-15

**Authors:** Jihua Yang, XiaoHong Wei, Fang Hu, Wei Dong, Liao Sun

**Affiliations:** 1 Department of Endocrinology and Metabolism, The Fifth Affiliated Hospital of Sun Yat-sen University, Zhuhai, 519000 China; 2 Department of Pathology, Eastern Hepatobiliary Surgery Hospital, The Second Military Medical University, Shanghai, China

**Keywords:** Pancreatic adenocarcinoma, Ferroptosis, Prognosis, Bioinformatics, Genes

## Abstract

**Background:**

Molecular markers play an important role in predicting clinical outcomes in pancreatic adenocarcinoma (PAAD) patients. Analysis of the ferroptosis-related genes may provide novel potential targets for the prognosis and treatment of PAAD.

**Methods:**

RNA-sequence and clinical data of PAAD was downloaded from The Cancer Genome Atlas (TCGA) and Gene Expression Omnibus (GEO) public databases. The PAAD samples were clustered by a non-negative matrix factorization (NMF) algorithm. The differentially expressed genes (DEGs) between different subtypes were used by “limma_3.42.2” package. The R software package clusterProfiler was used for functional enrichment analysis. Then, a multivariate Cox proportional and LASSO regression were used to develop a ferroptosis-related gene signature for pancreatic adenocarcinoma. A nomogram and corrected curves were constructed. Finally, the expression and function of these signature genes were explored by qRT-PCR, immunohistochemistry (IHC) and proliferation, migration and invasion assays.

**Results:**

The 173 samples were divided into 3 categories (C1, C2, and C3) and a 3-gene signature model (ALOX5, ALOX12, and CISD1) was constructed. The prognostic model showed good independent prognostic ability in PAAD. In the GSE62452 external validation set, the molecular model also showed good risk prediction. KM-curve analysis showed that there were significant differences between the high and low-risk groups, samples with a high-risk score had a worse prognosis. The predictive efficiency of the 3-gene signature-based nomogram was significantly better than that of traditional clinical features. For comparison with other models, that our model, with a reasonable number of genes, yields a more effective result. The results obtained with qPCR and IHC assays showed that ALOX5 was highly expressed, whether ALOX12 and CISD1 were expressed at low levels in tissue samples. Finally, function assays results suggested that ALOX5 may be an oncogene and ALOX12 and CISD1 may be tumor suppressor genes.

**Conclusions:**

We present a novel prognostic molecular model for PAAD based on ferroptosis-related genes, which serves as a potentially effective tool for prognostic differentiation in pancreatic cancer patients.

**Supplementary Information:**

The online version contains supplementary material available at 10.1186/s12935-021-02431-8.

## Background

Pancreatic cancer has a low 5-year survival rate and is one of the cancers that have high mortality [1]. There were 458,918 new pancreatic cancer cases and 432,242 deaths due to it, reported globally in 2018 [2]. Adenocarcinoma is the most common type of pancreatic cancer and accounts for > 90% of the diagnosed pancreatic cancer cases. Although adjuvant chemotherapy and other multimodal treatments have been developed, surgery is still the most effective method for treating this disease [3]. Despite advances in the treatment of pancreatic adenocarcinoma (PAAD), the 5-year survival rate remains only 9% [4]. To tackle this, there is an urgent need to identify the prognostic biomarkers of PAAD. This would aid clinicians to predict the clinical outcomes promptly and accurately as well as initiate a protocol for a personalized treatment regimen.

Ferroptosis, a type of cell death, plays a vital role in inhibiting tumorigenesis by removing cells that either has a deficiency or overabundance of key nutrients or cells damaged by environmental pressure [5]. Unlike autophagy and apoptosis, ferroptosis is an iron (Fe) and reactive oxygen species (ROS)-dependent form of cell death, [6]. It regulates cell death through the overproduction of phospholipid hydroperoxides in a mechanism different from that of autophagy and apoptosis. It is induced in abnormal cells due to loss of the selective permeability of the plasma membrane and oxidative stress caused by intense membrane lipid peroxidation [7]. Ferroptosis plays an important regulatory role in the occurrence and progression of tumors and provides a promising therapeutic strategy for PAAD [8, 9].

Recently, research has focused on the immune infiltration and its role in cancer. Previous studies have demonstrated that infiltrating immune cells can be isolated from tumors suggesting that tumor immune infiltration is a crucial biological process of cancers [10]. Further, many studies indicate that ferroptosis regulators play an important role in immune environment of cancers including breast cancer [11], hepatocellular carcinoma [12], and others. However, the ferroptosis related genes of tumor immune infiltration in PAAD remain largely unknown. Based on the essential roles of the ferroptosis related genes within the tumor immune infiltration in carcinogenesis and progression could have major potential as biomarkers in PAAD.

With the development of next-generation sequencing technology, gene transcription profiles in PAAD can be better understood. Based on The Cancer Genome Atlas (TCGA) and the comprehensive Gene Expression Omnibus (GEO) databases, many genes be found contribute to the development of PAAD. Recent studies have shown that CA9, CXCL9, and GIMAP7 genes specifically regulate the expression of FoxO1, thereby regulating immune infiltration in PAAD [13]. Further, FOXP4-AS1 exerts oncogenic activity in PAAD [14] and COL11A1 as an immune infiltrates correlated prognosticator in pancreatic adenocarcinoma [15].

In this study, we identified ferroptosis-associated genes and constructed molecular subtypes of PAAD based on TCGA and GEO databases. Finally, we established a 3-gene signature prognostic model and verified its ability to predict the prognostic risk and immune infiltrates of PAAD. This serves as a potentially effective tool for prognostic risk prediction in patients with pancreatic cancer.

## Methods

### Data source and processing

Expression data and corresponding clinical follow-up information from the TCGA-PAAD data set were downloaded using the UCSC genome browser database. GSE62452 chip data sets with survival time were selected from Gene Expression Omnibus (GEO) database. The GSE62452 data set is Microarray gene-expression profiles of 69 pancreatic tumors and 61 adjacent non-tumor tissue from patients with pancreatic ductal adenocarcinoma. ([HuGene-1_0-st] Affymetrix Human Gene 1.0 ST Array [transcript (gene) version]). Henceforth, samples that lacked clinical follow-up information were removed and the expression of multiple gene symbols was considered the median value.

### Molecular subtype identification using the non-negative matrix factorization algorithm

Firstly, 60 ferroptosis-related genes were retrieved from the literature [15–18] (Table 1). Next, 58 ferroptosis-related genes with gene expression data were matched with the TCGA-PAAD data set, and PAAD samples were clustered by non-negative matrix factorization (NMF). The standard "Lee" was selected in the NMF method, and ten iterations were performed. The cluster number ‘k’ was set at 2–10, the average contour width of the common member matrix was determined by the R package "NMF", and the samples were divided into three categories.

**Table 1:** The clinical statistical information of the samples

Clinical features	Train	Test	GSE15048
OS			
0	39	43	16
1	49	42	50
Grade			
G1	16	11	
G2	48	44	
G3	23	27	
G4	1	3	
Gender			
Male	39	40	
Female	49	45	
Age			
≤ 48	6	6	
> 48	82	79	
M_stage			
M0	38	38	
M1	5	0	
MX	45	44	
N_stage			
N0	25	24	
N1	63	61	
T_stage			
T1	3	3	
T2	12	11	
T3	72	69	
T4	1	2	
Stage			
Stage i	10	9	
Stage ii	74	72	
Stage iii	1	2	
Stage iv	3	2	

### Identification and functional analysis of differentially expressed genes (DEGs)

The limma_3.42.2 package [19] was used to analyze the differentially expressed genes (DEGs) in cluster 1, cluster 2, and cluster 3 among the molecular subtypes, based on the threshold false discovery rate (FDR) < 0.05 and |log2FC|> 0.5 filters. The DEGs shared by the three clusters were identified, and the Kyoto Encyclopedia of Genes and Genomes (KEGG) pathway analysis and gene ontology (GO) functional enrichment analysis were performed on them through the R package clusterProfiler (v3.16.1) (Additional file 1).

### Comparative analysis of immune scores among molecular subtypes

The single-sample gene set enrichment analysis (ssGSEA) method of the GSVA (Gene Set Variation Analysis) package was used to identify the immune score-based relationships among the molecular subtypes in the TCGA-PAAD data set. The scores of 28 immune cells were assessed [20] and then the differences in immune scores among the molecular subtypes were compared.

### Training set and internal test set construction

A total of 173 samples in the TCGA-PAAD data set were divided into a training set and a test set. To prevent the random allocation bias from affecting the stability of subsequent modeling, all samples were put back into random grouping, two hundred times in advance. Using the training data set, a univariate Cox proportional risk regression model was constructed for ferroptosis-related genes (n = 60) and survival data was constructed using the coxph function with survival R package. Since 58 ferroptosis-related genes had expression profile data in our data set, only these were selected for the univariate Cox regression analysis, and P < 0.05 was selected as the threshold for filtering. The R software package “glmnet_4.10–1” [21] was used to carry out the LASSO Cox regression analysis. We first analyzed the changing trajectory of each independent variable, and later used the fivefold cross-validation to build a model and analyze the confidence interval under each lambda. The target genes were selected by multivariate Cox regression analysis, and a prognostic Kaplan–Meier (KM) curve was established.

### 3-Gene signature robustness in different data sets

The risk scores of each sample were calculated separately based on the expression level of the sample. KM-curve analysis showed significant differences between the high and low expression groups. Furthermore, we used the R software package timeROC_0.4 [22] to conduct ROC analysis of the prognostic classification of the risk score and analyze the prognostic classification efficiency at 1-year, 3-years, and 5-years. The model and the survival coefficient, developed using the training dataset, were adopted to evaluate the entire TCGA-PAAD data set, calculate the risk score of each sample and establish the risk score distribution of the samples. The independent GSE62452 data set was used to analyze the robustness of the model.

### Univariate and multivariate analyses of the 3-gene signature

To identify the independence of the 3-gene signature model in clinical applications, we performed Cox regression analysis on the TCGA-PAAD training dataset. Based on the results of univariate and multivariate analyses, we used the TCGA-PAAD training dataset to construct a histogram. In addition, corrected curves were used to analyze the prediction accuracy of nomogram at 1, 3, and 5 years.

### Tissue samples

PAAD tissues were derived from surgically resected specimens and snap-frozen in liquid nitrogen until RNA extraction. None of the patients received chemotherapy or radiation therapy before surgery. All patients signed informed consent forms provided by the Eastern Hepatobiliary Surgery Hospital. This study was approved by the Ethics Committee of the Eastern Hepatobiliary Surgery Hospital.

### RNA isolation and RT-qPCR analysis

RNA was extracted from tissues using the TRIzol reagent (Invitrogen, Carlsbad, CA, USA) and was reverse-transcribed into cDNA using the QuantiTect Reverse Transcription Kit (Qiagen, Valencia, CA, USA). Quantitative PCR (qPCR) uses real-time fluorescence to measure the quantity of DNA present at each cycle during a PCR. Real-time qPCR analyses were quantified with SYBR-Green (Takara, Otsu, Shiga, Japan), and expression levels were normalized to GAPDH levels.

### Immunohistochemistry

Immunohistochemistry was performed by two-step method according to the instructions (PV-9000; ZSGB-BIO, Beijing, China). Pancreatic cancer samples were fixed in 10% formalin, embedded in paraffin, and processed into 5-µm sequential sections. The samples were de-waxed with ethanol and blocked to inhibit the endogenous peroxidase activity. After this, samples were heated in a microwave for antigen retrieval, cooled to room temperature, and blocked using goat serum for 30 min at 37 °C. The samples were incubated overnight at 4 °C with rabbit anti-ALOX5 (ab169755), anti-ALOX12 (ab211506), and anti-CISD1 (ab203096) (Abcam, USA) (1:200), followed by incubation with horseradish peroxidase-coupled goat anti-rabbit secondary antibody (PV-9000; ZSGB-BIO, Beijing, China) at 37 °C for 30 min. The samples were then stained with 3,3′-Diaminobenzidine (DAB). Cell nuclei were stained blue with hematoxylin. The sections were then dehydrated, cleared with xylene, and mounted. ALOX5, ALOX12, and CISD1 expressions were determined by immunohistochemistry (IHC) using the streptavidin peroxidase method, with adjacent tissues serving as the controls. The experimental procedure was performed as per the manufacturer’s instructions. Image-Pro Plus 6.0 Software (Media Cybernetics, USA) was used to analyze protein expression and perform statistical analysis of the results obtained by IHC.

### Cell culture and transfection

The human PAAD cell line T3M4 and Panc 02.03 were provided by the National Collection Authenticated Cell Cultures (Shanghai, China). The T3M4 cell lines are derived from the metastatic lymph node tissue of human pancreatic cancer and are epithelial-like cells. The T3M4 cell lines were cultured in DMEM (Dulbecco’ modified eagle medium) (Gibco, Grand Island, NY, USA). Supplemented with 10% fetal bovine serum (Invitrogen, San Diego, CA, USA) at 37 °C under 5% CO_2_ in a humidified incubator. The Panc 02.03 cell lines are derived from human primary pancreatic cancer and are epithelial-like cells. The Panc 02.03 cell lines were cultured in RPMI-1640 (Gibco, Grand Island, NY, USA) with 15% fetal bovine serum at 37 °C under 5% CO_2_ in a humidified incubator. Si-ALOX5 (No: CAT#: SR319325) was purchased from Origene (Beijing, China). Transfection was performed using Lipofectamine 3000 reagent (No. L3000015, Invitrogen, China) according to the instructions and cell transfection efficiency was 82%.The human ALOX12 and CISD coding sequences were cloned into the pEZ-M03 Vector.

### Cell viability assays

The si-ALOX5 transfected in T3M4 cell lines, and ALOX12 or CISD transfected in Panc 02.03 cell lines. Forty-eight hours post infection, the cells were collected and seeded into 96-well plates at a concentration of 2000 cells per well. Cell viability was detected by Cell Counting Kit-8 assay (CCK-8, Dojindo, Japan) according to the manufacturer’s protocol after 48 h. Te absorbance at 450 nm was measured using an automatic microplate reader (BioTek, Winooski, VT, USA). All Cell Counting Kit-8 assay were performed in five times.

### Cell migration and invasion assays

The si-ALOX5 transfected in T3M4 cell lines, and ALOX12 or CISD transfected in Panc 02.03 cell lines. Forty-eight hours post infection, the cells were collected. For the migration assay, 800 μl DMEM with 20% serum was added to the lower chamber of a Transwell plate (Corning, NY, USA), and 1.5 × 10^5^ cells were added in the upper chamber. The cells were harvested, resuspended in serum-free media and placed into the upper chamber of a Transwell membrane filter (Corning, NY, USA) for the migration assays or in the upper chamber of a transwell membrane filter coated with Matrigel (Corning) for the invasion assays.The T3M4 and Panc 02.03 cells were added in the upper chamber. After incubation for 24 h at 37 °C, the Transwell chamber was removed. The cells were stained with methanol and 0.1% crystal violet, imaged, and the relative cell density was measured by ImageJ (National Institute of Health, USA). ImageJ software was used to analyze and calculate the migration and invasion area of the cells. The migration and invasion index (%) depicts a proportion of an area where cells have invaded in percentage and is calculated as epithelium area divided by the total area of invaded cells area. The area describes the overall area (μm^2^) of invaded cells. Evaluation of invasive capacity was performed by counting invading cells under a microscope (40 × 10). Five random fields of view were analyzed for each chamber. All cell migration and invasion assays were performed in five times.

### Statistical analysis

All data were analyzed using the SPSS 21.0 statistical software program (IBM Corporation, Armonk, NY, USA). Graphs were generated with GraphPad Prism 8.0 software (GraphPad Software, Inc., San Diego, CA, USA). Student’s t-tests were performed. For a two-tailed t-test, P < 0.05 was essential for considering the results to be statistically significant.

## Results

### Sample information statistics

After data preprocessing, there were 173 samples in TCGA-PAAD, with 88 samples in the training set and 85 samples in the test set. GSE62452 included 66 samples. The clinical statistical information of the samples is shown in Table 1.

### Identification of three molecular subtypes

**Fig. 1:**
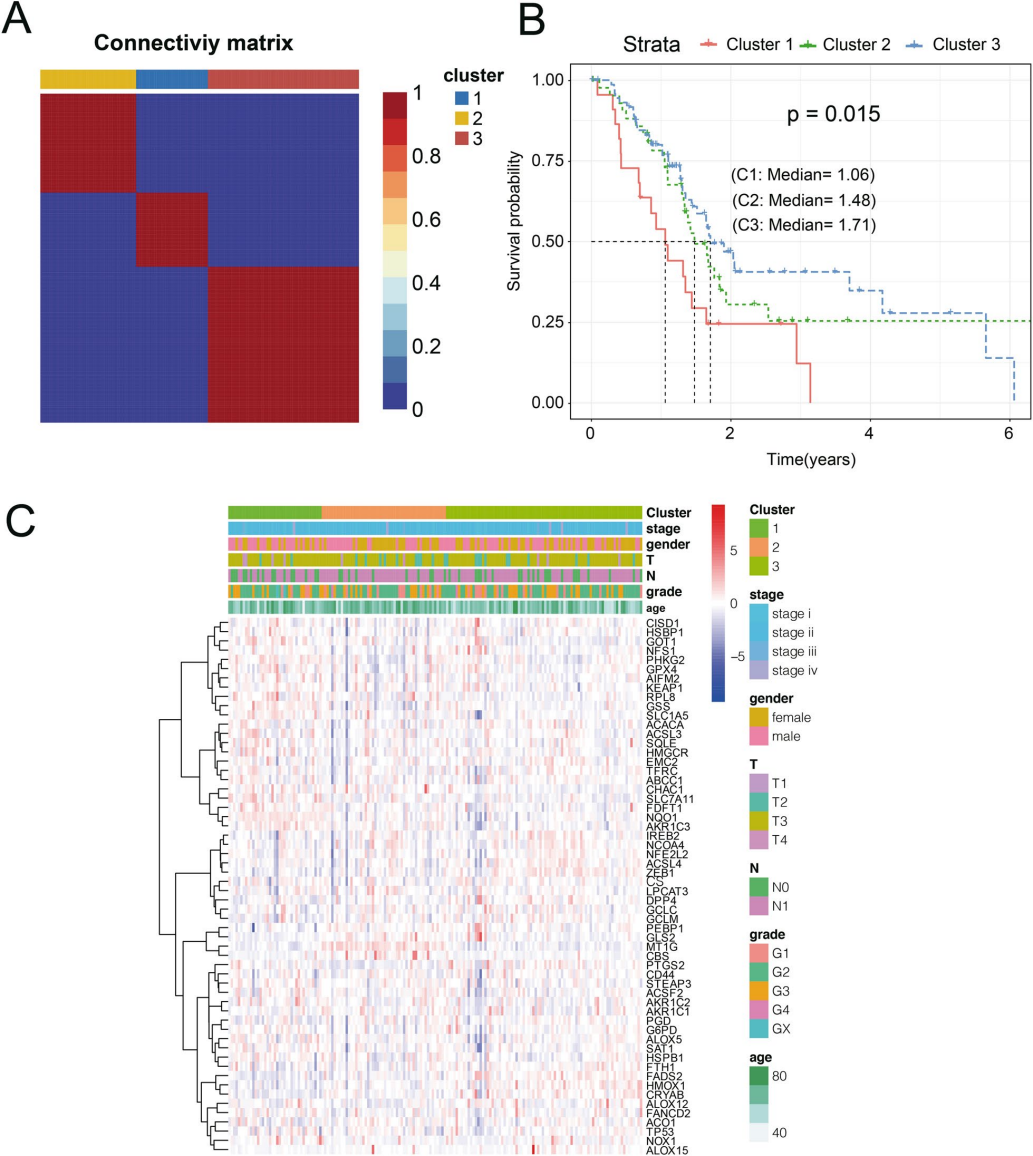
The samples were divided into 3 categories. **A** The expression of prognostic ferroptosis-related genes in the three categories. **B** The prognostic relationships showed significant differences in C1, C2, and C3. **C** Ferroptosis-related genes in TMN staging, stage, grade, age and different clusters

The 173 samples were divided into 3 categories: C1, C2, and C3. The expression of prognostic ferroptosis-related genes in the three categories is shown in Fig. 1A. The expression levels of these genes are different in the 3 subtypes and that most of the genes are highly expressed in the C1 subgroup. Further analysis of the prognostic relationships between the two groups showed significant differences in C1, C2, and C3 (Fig. 1B, log-rank test, P < 0.05). We analyzed the expression patterns of ferroptosis-related genes in TMN staging, Stage, Grade, Age and different Clusters, as shown in heat map (Fig. 1C).

### Analysis of DEGs among subtypes

**Fig. 2:**
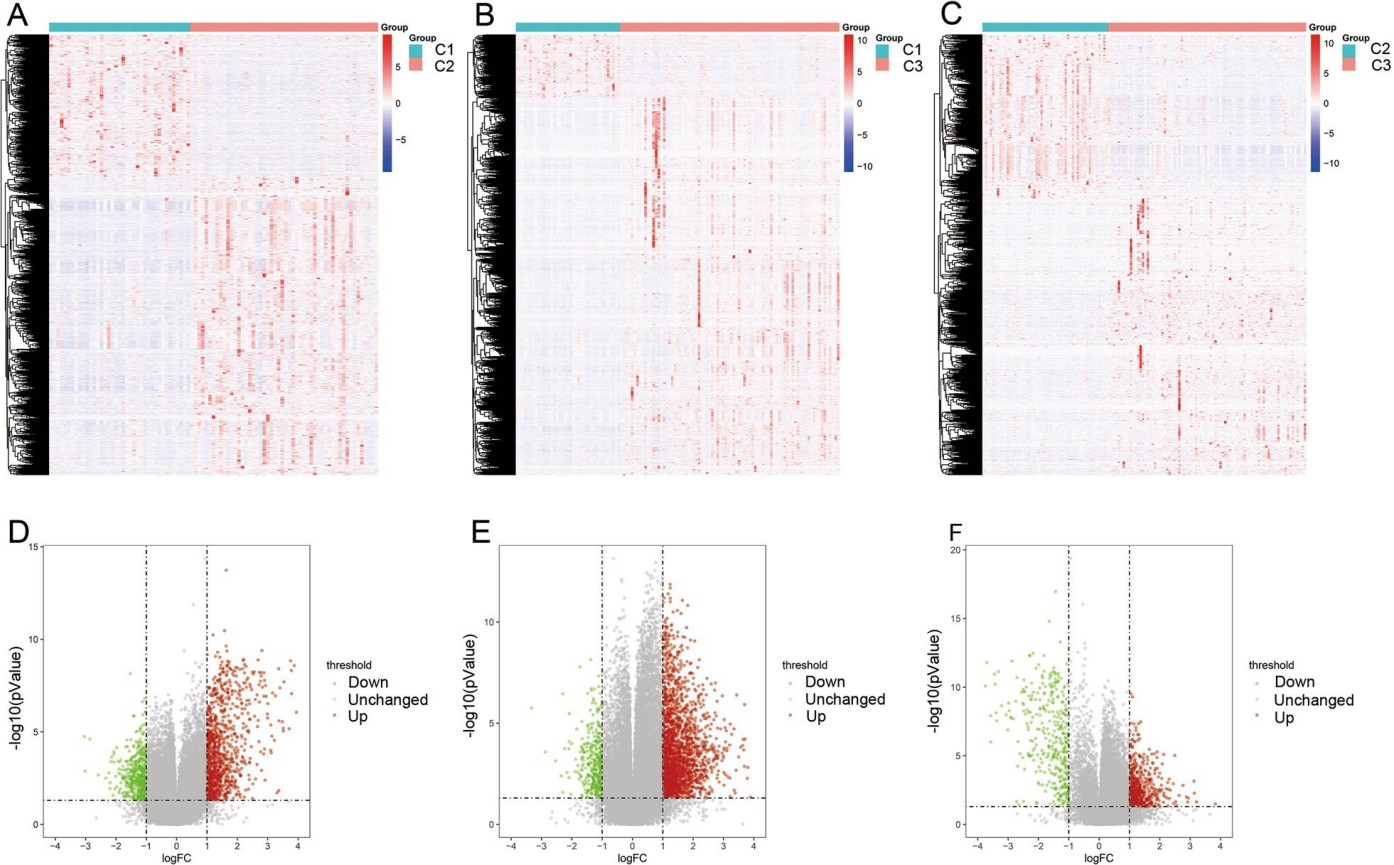
The differentially expressed genes among subtypes. **A**, **D** 4903 DEGs were identified between Cluster 1 and Cluster 2. **B**, **E** 6572 DEGs between Cluster 1 and Cluster 3. **C**, **F** Between Cluster 2 and Cluster 3 there were 3473 DEGs

With the help of the limma_3.42.2 package, 4,903 DEGs were identified between Cluster 1 and Cluster 2, among which 2,717 genes were up-regulated and 2,186 genes were down-regulated (Fig. 2A, D). There were 6,572 DEGs between Cluster 1 and Cluster 3, among which 4,963 genes were up-regulated and 1,609 genes were down-regulated (Fig. 2B, E). Between Cluster 2 and Cluster 3 there were 3,473 DEGs, among which 2633 genes were up-regulated and 840 genes were down-regulated (Fig. 2C, F) (Additional file 1: Table S1). After taking the intersection of all three clusters 230 genes were obtained.

**Fig. 3:**
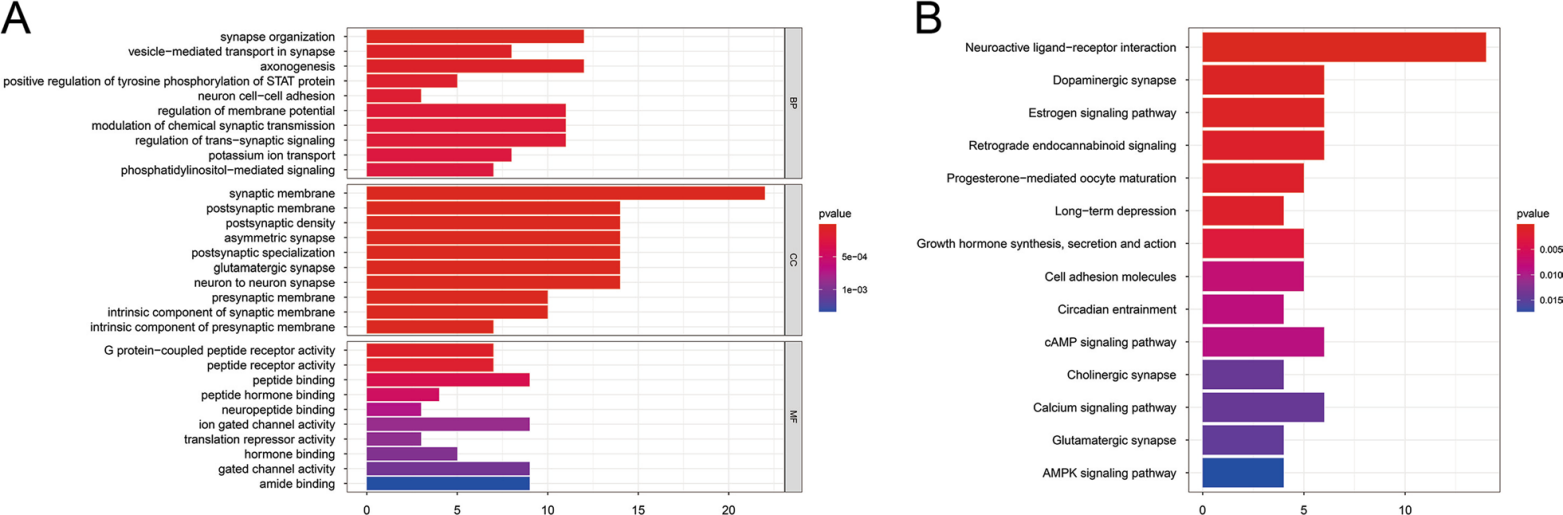
KEGG pathway analysis and GO functional enrichment analysis. **A** A total of 179 GO-BP pathways, 47 GO-CC pathways, and 67 GO-MF pathways were annotated. **B** 14 KEGG pathways were identified, 6 of which were significant

KEGG pathway analysis and GO functional enrichment analysis were performed on the 230 DEGs in the PAAD subtype group. A total of 179 GO-BP pathways, 47 GO-CC pathways, and 67 GO-MF pathways were annotated (Fig. 3A). 14 KEGG pathways were identified, 6 of which were significant (FDR < 0.05) (Fig. 3B). The detailed information is shown in Additional file 2.

### Comparative analysis of immune scores among molecular subtypes

**Fig. 4:**
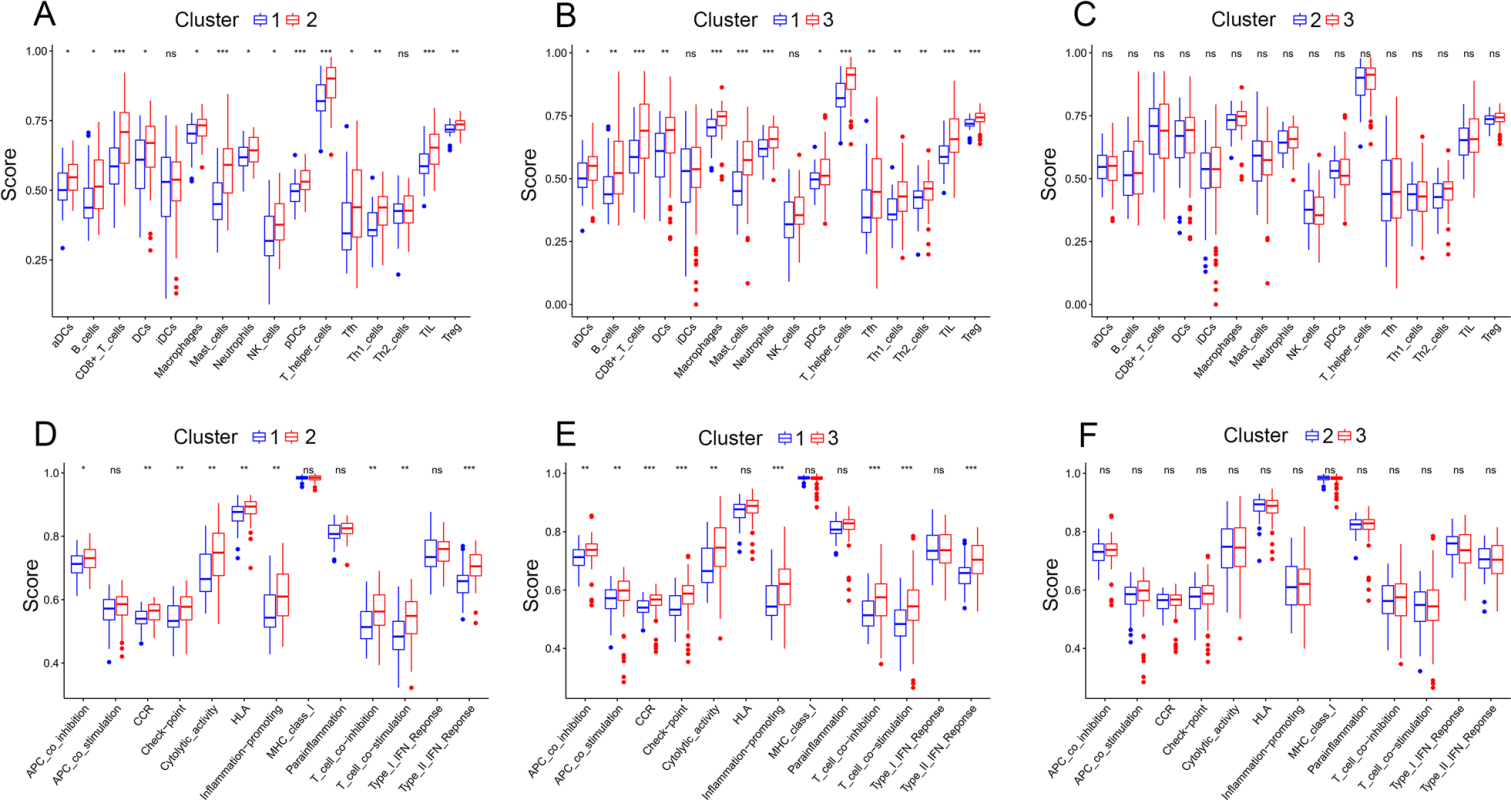
The relationship of the immune scores between the molecular subtypes. **A**, **B** The immune score of subtype C1 is lower than that of subtype C2 and C3. **C** The immune score of the C2 subtype is lower than the C3 subtype. **D**–**F** The box plot of differences in the scores of immune-related pathways among the three subtypes

In order to identify the relationship of the immune scores between the molecular subtypes in the TCGA-PAAD data set, we used the ssgsea method of the GSVA package to score 29 immune cells (16 are immune cells and 13 are immune-related pathways) (cell markers are from the reference [23]) and then compare the differences between immune cells and immune-related pathways in molecular subtypes. The results shown that: the immune score of subtype C1 is lower than that of subtype C2 (Fig. 4A); the immune score of subtype C1 is lower than the C3 subtype (Fig. 4B); the immune score of the C2 subtype is lower than the immune score of the C3 subtype (Fig. 4C). At the same time, we have drawn a box plot of differences in the scores of immune-related pathways among the three subtypes (Fig. 4D–F). Combined with the prognostic survival curve in Fig. 1B, it can be seen that the C1 subtype has the worst prognosis, and the C3 subtype has the best prognosis. A higher immune infiltration score is often accompanied by a better prognosis.

### Construction of a 3-gene signature

**Fig. 5:**
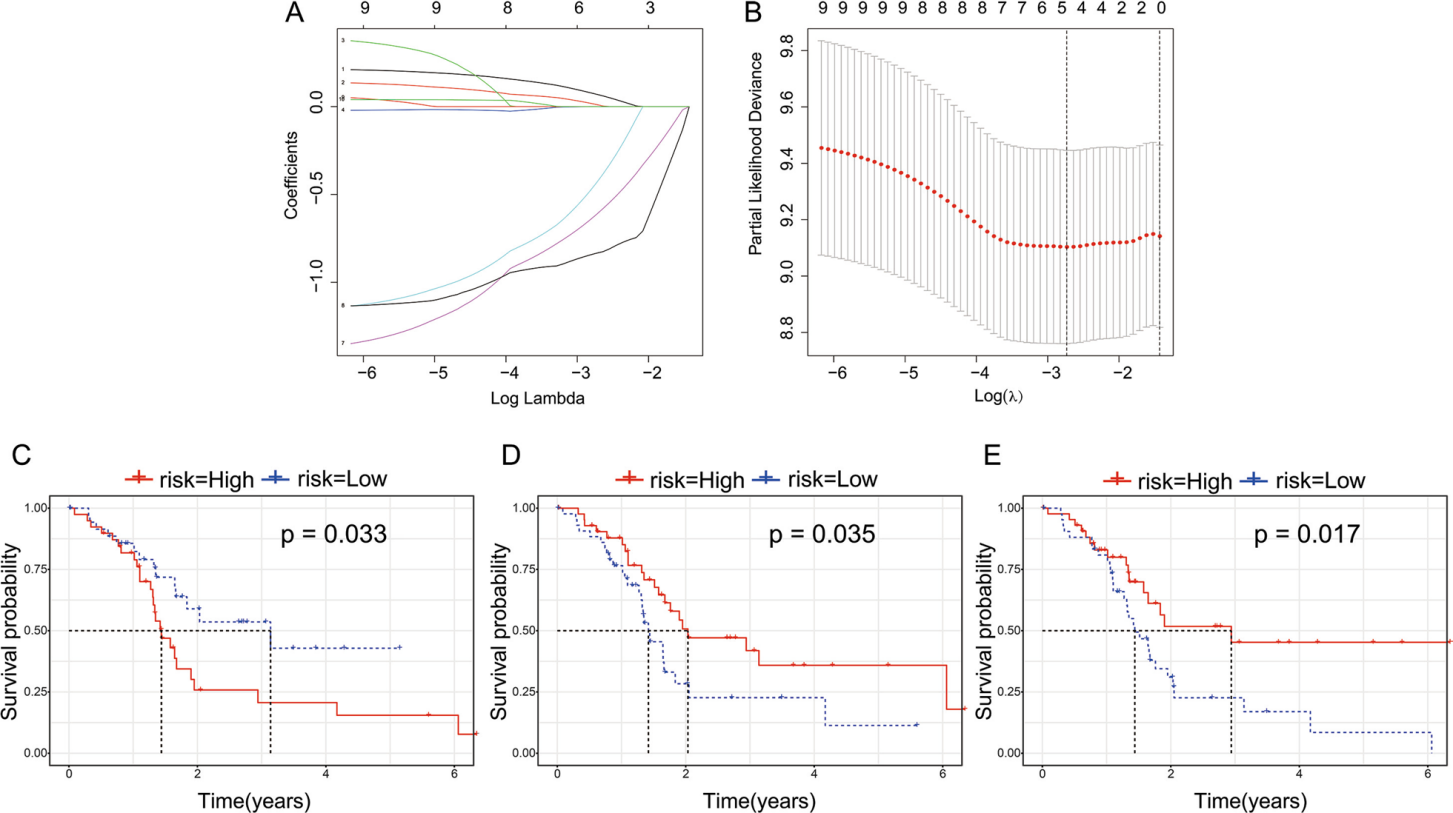
Constructed a 3-gene signature. **A** The changing trajectory of each independent variab. **B** The confidence interval under each lambda. **C**–**E** Prognostic KM-curves of the three genes

The R package survival coxph function is used to perform a univariate Cox proportional hazard regression model for ferroptosis-related genes (n = 60) and survival data in training set. Because only 58 ferroptosis-related genes are expressed in our expression profile data and we selected these genes for single-factor cox regression analysis, and selected P < 0.05 as the threshold for filtering. Finally, there were 10 genes related to prognosis. Next, LASSO regression was used to further compress the 10 genes to reduce the number of genes in the risk model. The changing trajectory of each independent variable is shown in Fig. 5A, which indicates that with a gradual increase in lambda, the number of independent variable coefficients approaching zero gradually increases. We used fivefold cross-validation to construct a model and analyze the confidence interval under each lambda (Fig. 5B). The figure indicates that the model reached the optimal value at lambda = − 3.75. Hence, we selected 5 genes at lambda = − 3.75 as target genes and further selected 3 genes (ALOX5, ALOX12, and CISD1) by multivariate Cox regression analysis. Prognostic KM-curves of the three genes are shown in Fig. 5C–E, and all three genes could significantly improve the performance of distinguishing between the low-risk groups (LRG) and high-risk groups (HRG) in the training sample (P < 0.05). The final model based on the 3-gene signature was as follows:

RiskScore = 0.289* ALOX5 + (− 1.359)*ALOX12 + (− 1.053)* CISD1.

**Fig. 6:**
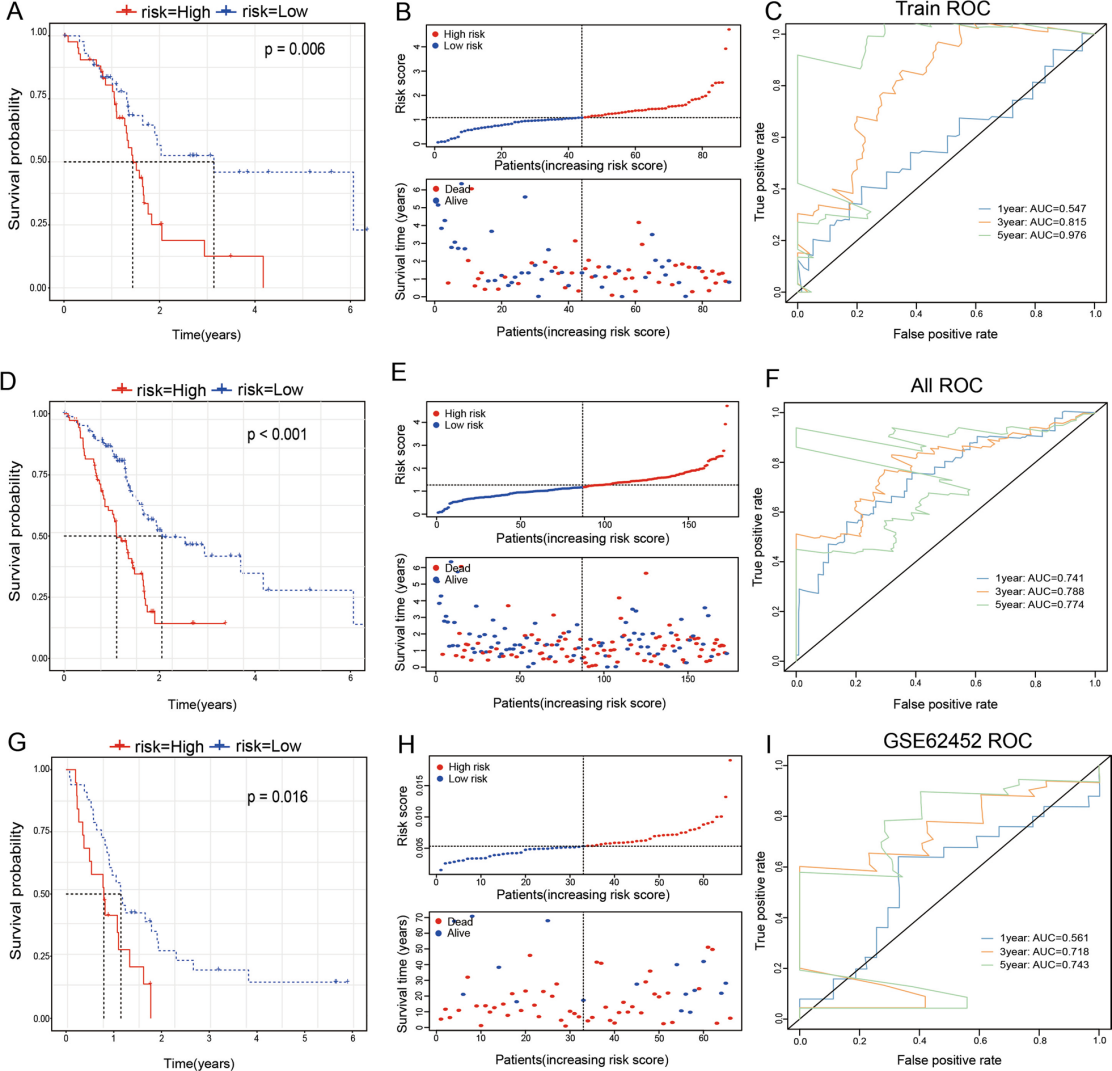
Calculated the risk score in three databases. **A**–**C** In TCGA-training set, KM-curve analysis; the risk of death for patients and ROC analysis were used for test the model. **D**–**F** In entire TCGA-PAAD dataset, KM-curve analysis; the risk of death for patients and ROC analysis were used for test the model. **G**–**I** In GSE62452 dataset, KM-curve analysis; the risk of death for patients and ROC analysis were used for test the model

Further, we calculated the risk score of each sample based on the expression level of the sample. In TCGA-training set, KM-curve analysis showed that there were significant differences between the high and low-risk groups (Fig. 6A, P = 0.006). The Fig. 6B indicates that the risk of death for a patient with a high-risk score was significantly higher than that of a patient with a low-risk score. This suggests that a sample with a high-risk score shows a worse prognosis. The model had a very high AUC (AUC for 1 year = 0.547, AUC for 3 years = 0.815, AUC for 5 years = 0.976) (Fig. 6C). The risk score distribution of the entire TCGA-PAAD dataset is shown in Fig. 6D–F, which also indicates that samples with a high-risk score had a worse prognosis.

Analysis of the GSE62452 showed that the risk score distribution was consistent with that of the training set and that the high-risk score samples had a worse prognosis (Fig. 6G–I). KM-curve analysis showed that there were significant differences between the high and low-risk groups. Analysis of the 1-year, 3-years, and 5-years prognostic prediction classification efficiencies indicated that the model had relatively high AUC at 3 years and 5 years (0.718 and 0.743, respectively).

### Risk model analysis and model comparison

**Fig. 7:**
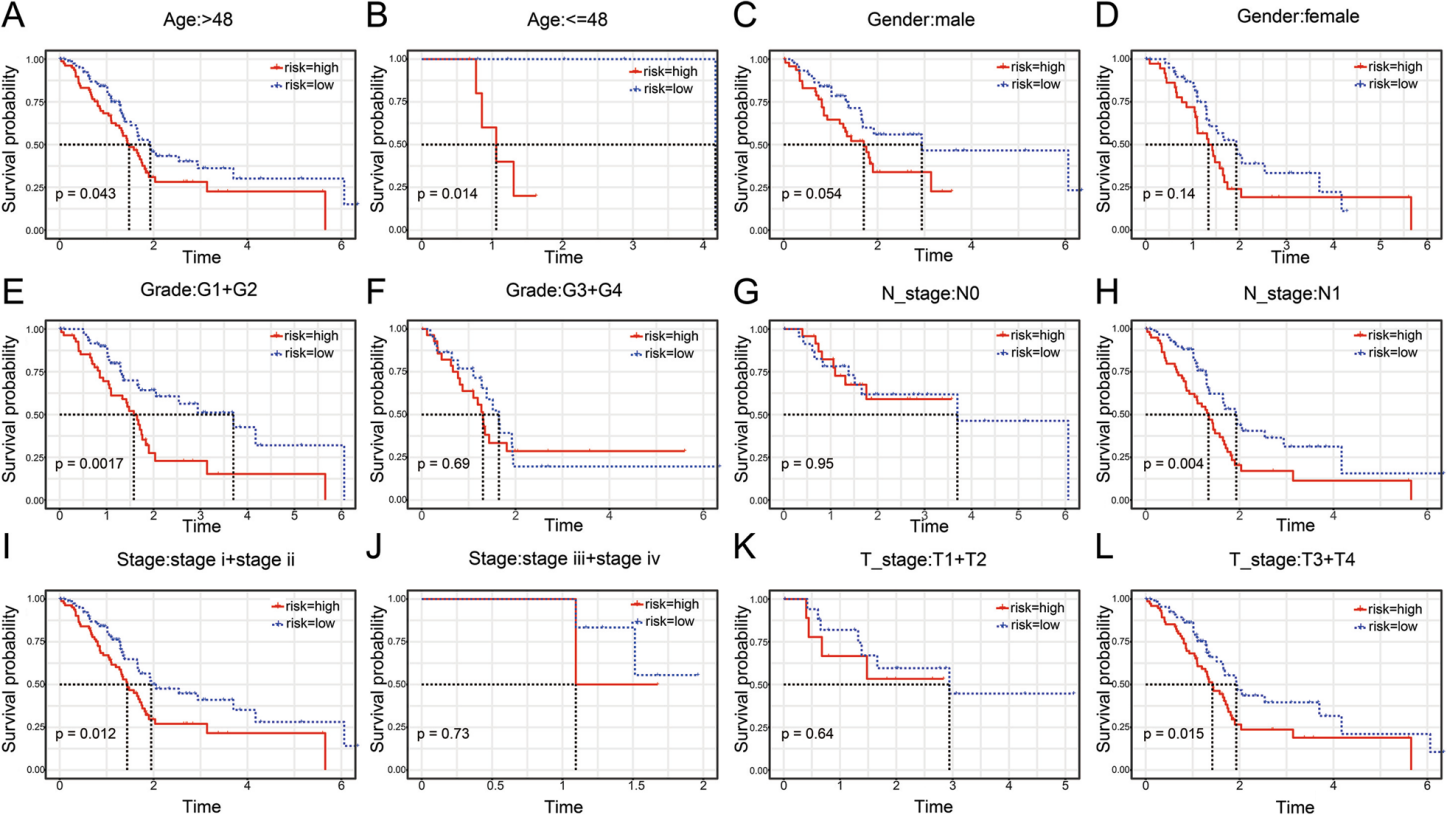
The low- and high-risk groups according to (**A**, **B**) age, (**C**, **D**) sex, (**E**, **F**) grade, (**G**, **H**) N stage, (**I**, **J**) stage, or (**K**, **L**) T stage

**Fig. 8:**
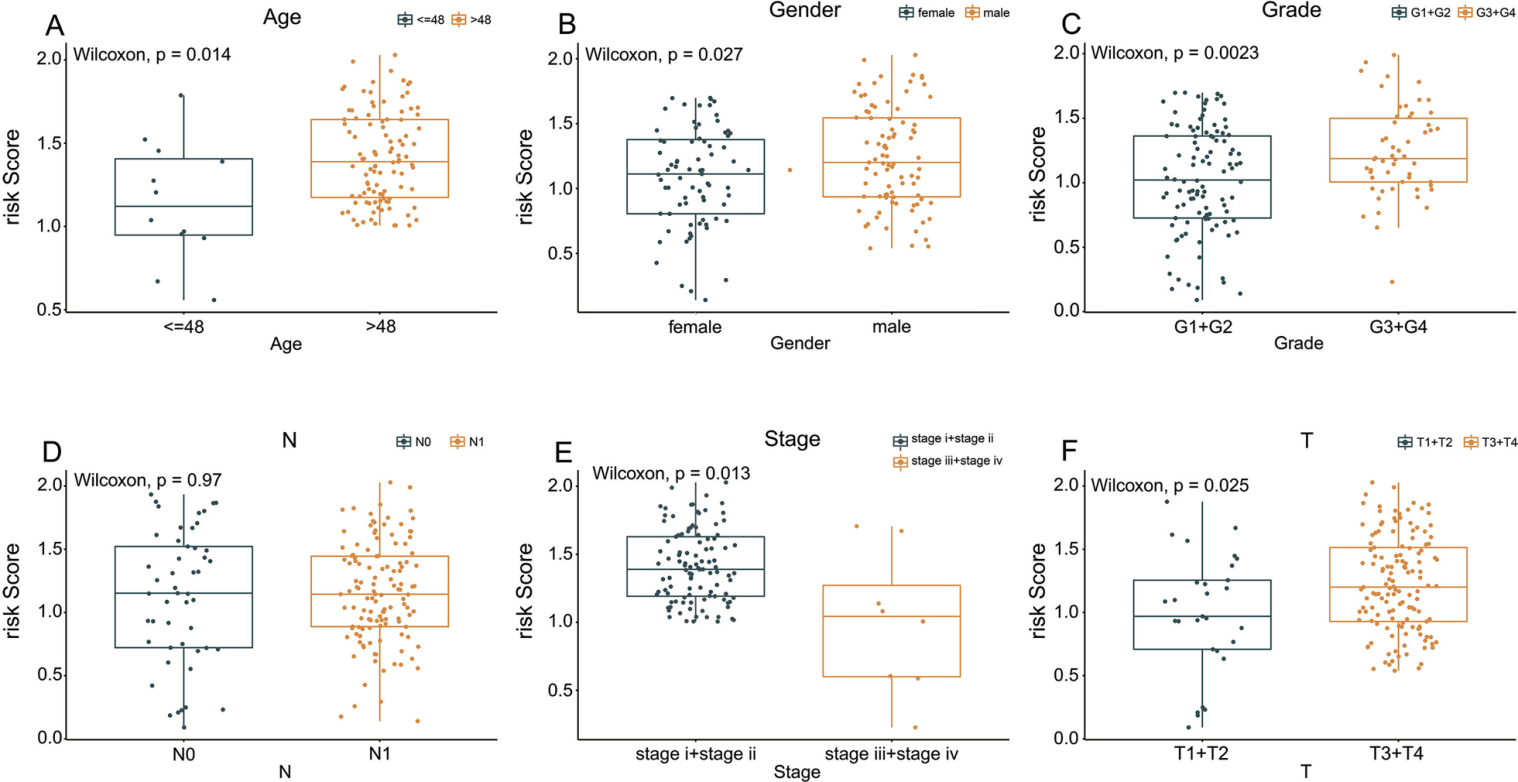
The risk score had a significant correlation with (**A**) age, (**B**) sex, (**C**) grade, (**D**) N stage, (**E**) stage, or (**F**) T stage

**Fig. 9:**
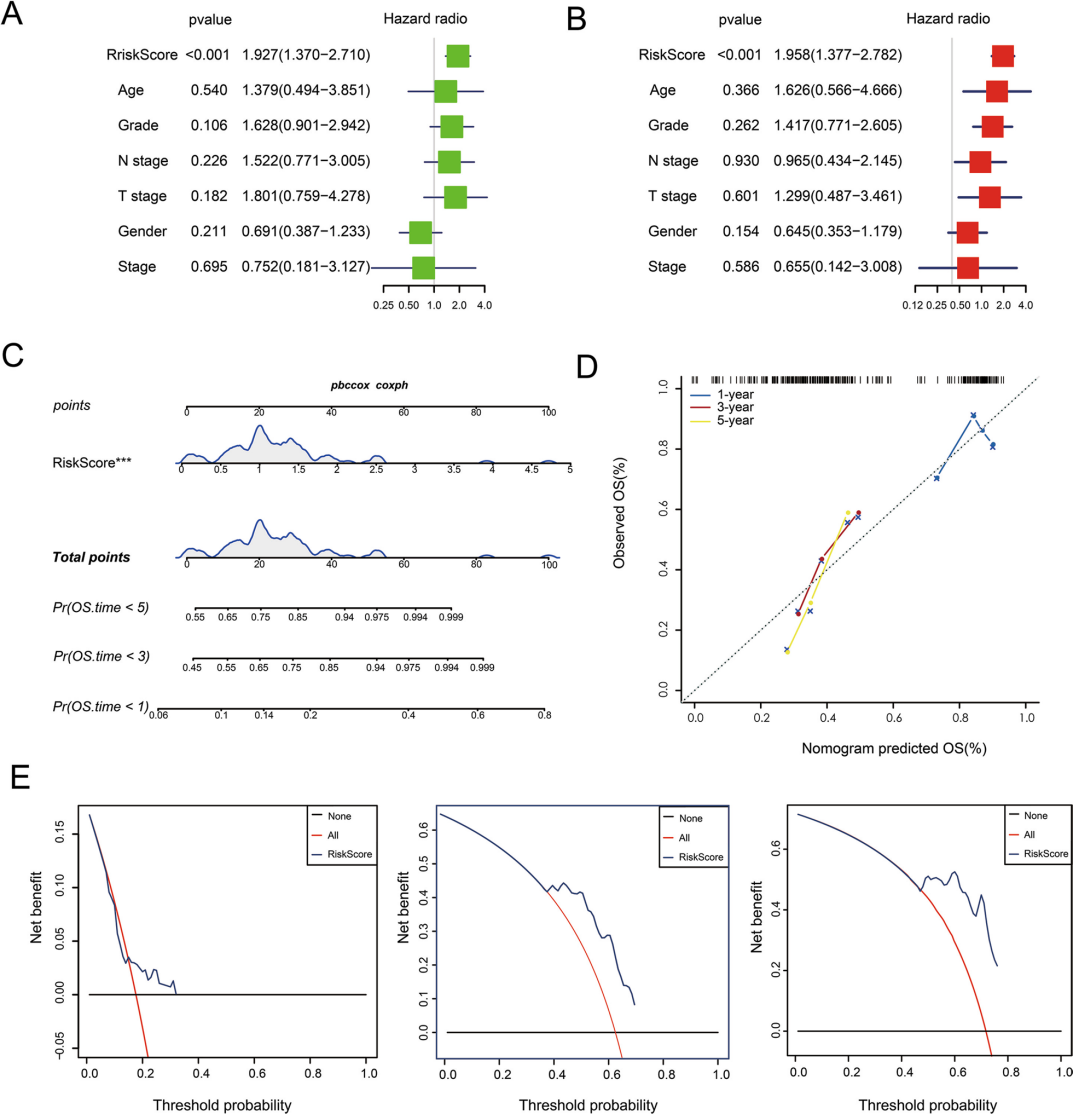
The 3-gene signature is an independent risk factor for prognosis in PAAD. **A** univariate and (**B**) multivariate Cox regression analyses. **C**, **D** The TCGA-PAAD training set was used to construct a nomogram. **E** The results of decision curve analysis (DCA) at 1-year, 3-years, and 5-years

Based on the 3-gene signature model, samples could be divided into low- and high-risk groups according to age, sex, grade, N stage, T stage, or clinical stage (Fig. 7A–L, P < 0.05). This further indicated that our model had good predictive power in different clinical subgroups. The risk score had a significant correlation with age, sex, grade, T stage, and clinical-stage but no significant correlation was with the N stage (Fig. 8A–F, P < 0.05). By univariate and multivariate Cox regression analyses, indicated that our model based on the 3-gene signature is an independent risk factor for prognosis in pancreatic cancer patients (Fig. 9A, B). Further, the TCGA-PAAD training set was used to construct a nomogram (Fig. 9C), which indicated that the risk model based on the 3 genes can accurately predict the prognosis of pancreatic cancer. In addition, we used corrected curves to analyze the prediction accuracy of the nomogram at 1, 3, and 5 years. The results indicated that the histogram had good prediction performance (Fig. 9D). Moreover, the results of decision curve analysis (DCA) at 1-year, 3-years, and 5-years (Fig. 9E) also indicated that the prediction efficiency of the histogram was good.

**Fig. 10:**
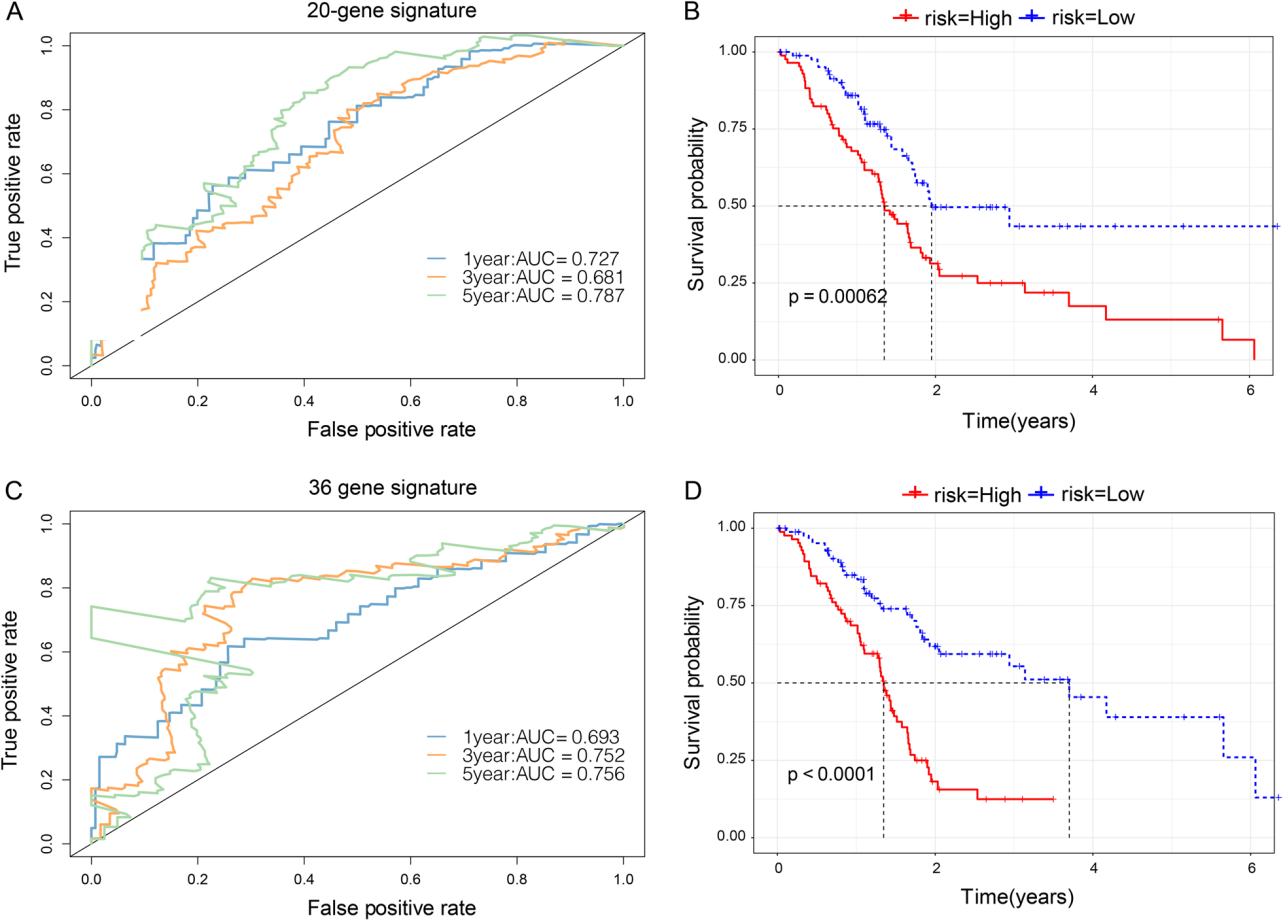
Comparison with other models. The PAAD prognosis of the high-risk score and low-risk score groups was more effective for (**A**, **B**) the 20-gene signature and (**C**, **D**) 36-gene signature models

For comparison with our model, we selected two prognostic risk models: 20 gene signatures [21] and 36 gene signatures [24]. Survival analysis indicated that the PAAD prognosis of the high-risk score and low-risk score groups was different except for the 20-gene signature (Fig. 10A, B) and 36-gene signature models (Fig. 10C, D) (log-rank P < 0.05). For the 36-gene signature model, the 1-, 3-, and 5-year AUC values were lower than our model. Moreover, the 1- and 3-year AUC values in the 20-gene signature were lower than our model. This proves that our model, with a reasonable number of genes, yields a more effective result.

### The expression and biological function of signature genes in PAAD

**Fig. 11:**
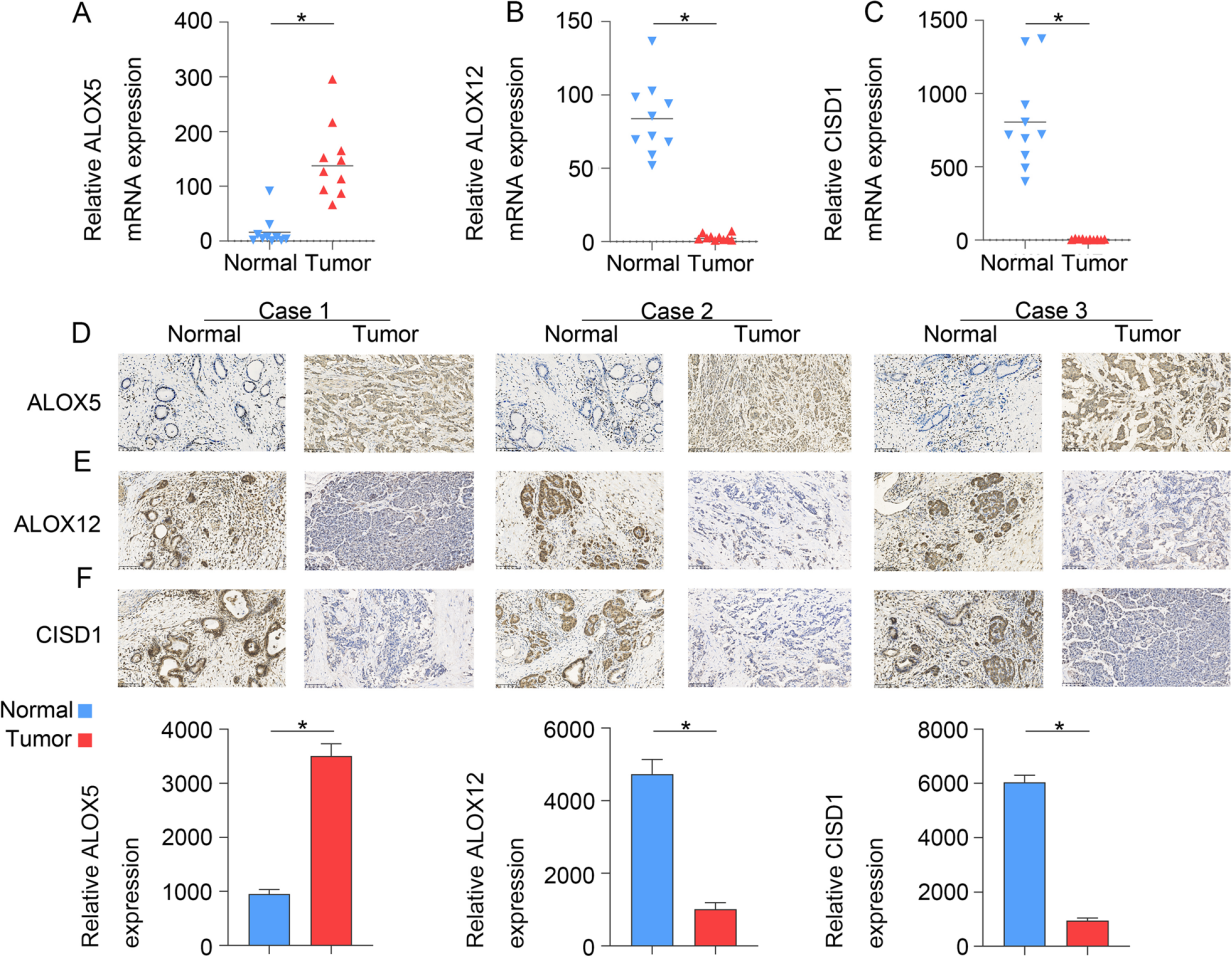
The expression of signature genes in PAAD.The results obtained with (**A**–**C**) qPCR and (**D**–**F**) IHC assays showed that ALOX5 was high expressed, ALOX12 and CISD1 were expressed at low levels in PAAD tissues

**Fig. 12:**
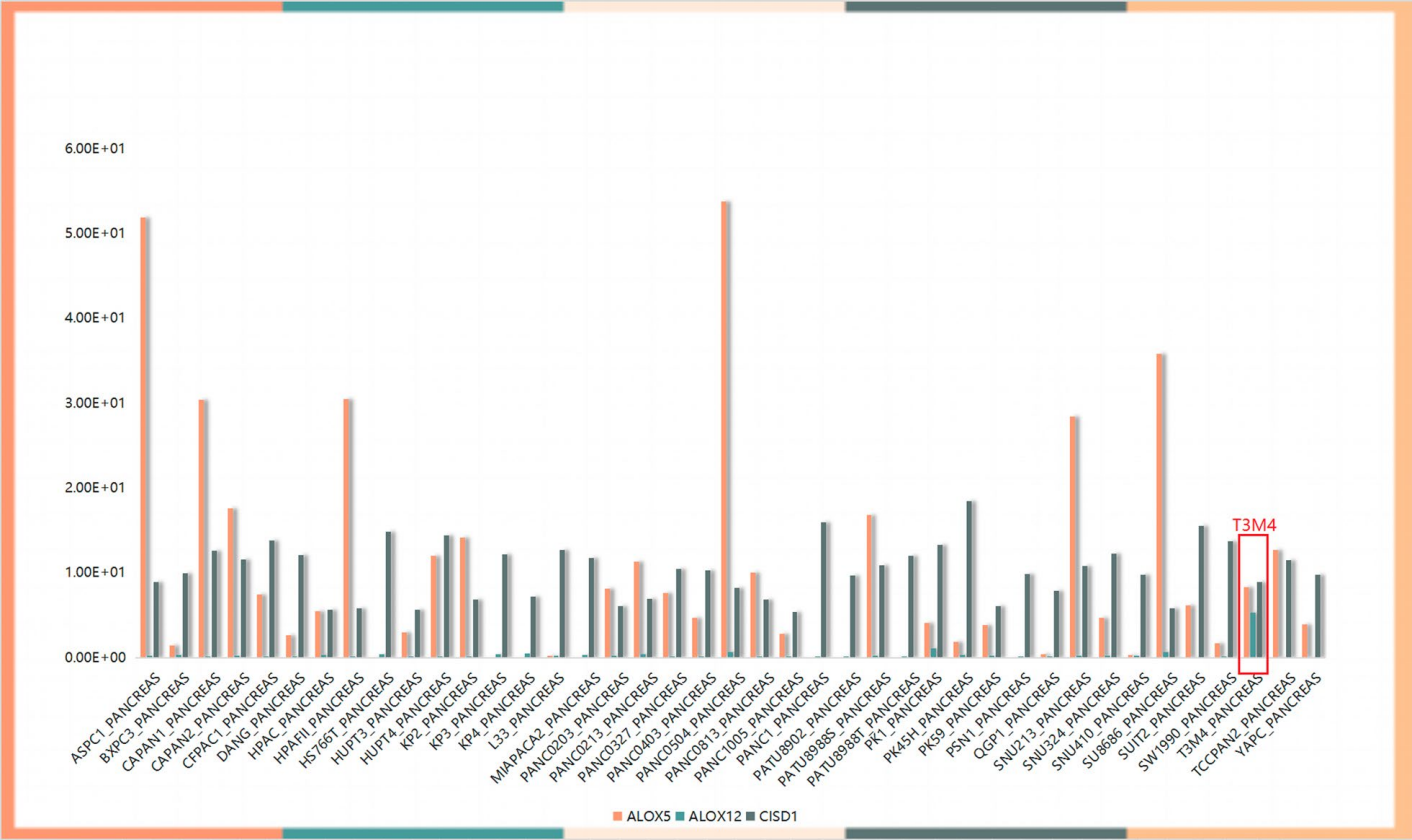
Cancer Cell Line Encyclopedia (CCLE) database to analyze the expression of ALOX5, ALOX12 and CISD1 in pancreatic cancer cells

**Fig. 13:**
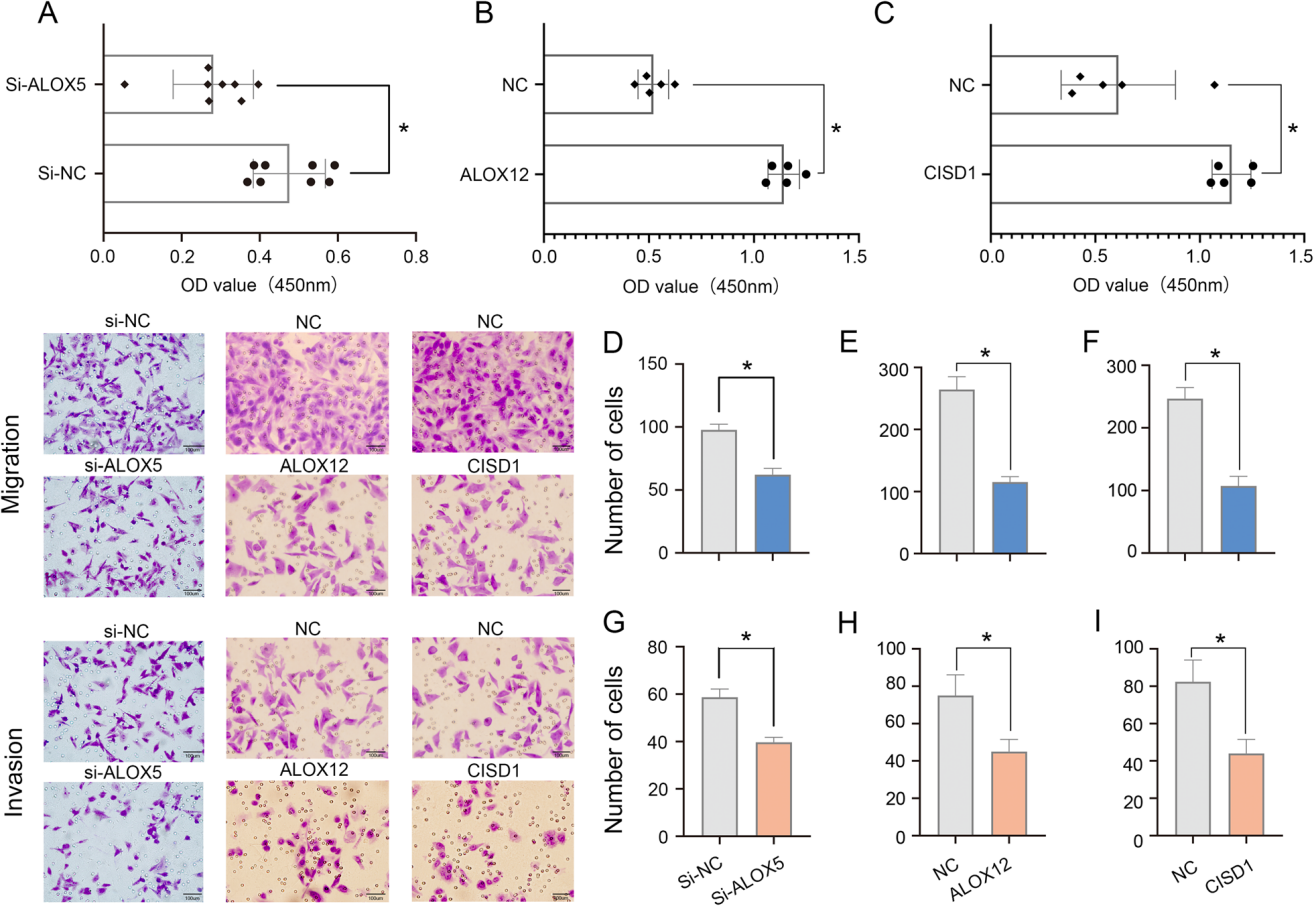
The function of signature genes in PAAD cells. Reduce ALOX5 expression significantly inhibited the (**A**) proliferation, (**D**) invasion and (**G**) migration ability of T3M4 cells, and up-regulation of ALOX12 and CISD1 expression suppressed the (**B**, **C**) proliferation, (**E**, **F**) migration and (**H**, **I**) invasion of Panc 02.03 cells

To verify that ALOX5 expression is upregulated and investigate whether ALOX12 and CISD1 are downregulated in pancreatic cancer tissue, 10 pancreatic cancer tissue specimens were tested. The results obtained with qPCR (Fig. 11A–C) and IHC (Fig. 11D–F) assays showed that ALOX5 was highly expressed regardless of whether ALOX12 and CISD1 were expressed at low levels in these pancreatic cancer tissue samples. Clinical details of these 10 patients are contained in the Additional file. To clarify the functional role of signature genes in PAAD cells, we used Cancer Cell Line Encyclopedia (CCLE) database to analyze the expression of ALOX5, ALOX12 and CISD1 in pancreatic cancer cells, and found that the expression of ALOX5 was relatively highly expressed in T3M4 cells. Further, in order to explore the biological function of ALOX5 (Fig. 12). siRNA was used to reduce the expression of ALOX5 in T3M4 cells and we used overexpression strategy for ALOX12 and CISD1 to check the proliferation, invasion, and migration of Panc 02.03 cells. CCK8 and transwell assays were used to determine the proliferation, invasion and migration ability of T3M4 cells and Panc 02.03 cells. The results showed that reduced ALOX5 (Fig. 13A, D, G) expression significantly inhibited the proliferation, invasion and migration ability of T3M4 cells, and up-regulation of ALOX12 (Fig. 13B, E, H) and CISD1 (Fig. 13C, F, I) expression suppressed the proliferation, migration and invasion of Panc 02.03 cells.

## Discussion

Ferroptosis is an iron oxide-dependent form of regulated cell death (RCD). It is the link that connects metabolism, redox biology, and human health. Accumulating evidence indicates that ferroptosis can be triggered to treat cancer to eradicate aggressive malignancies that are resistant to conventional therapies [25]. It is characterized by the accumulation of ROS and lipid peroxidation products to lethal levels. Although ferroptosis plays an important role in maintaining the survival of normal cells and tissues, it has been increasingly recognized that some carcinogenic pathways are related to ferroptosis, making cancer cells vulnerable to ferroptosis death [26, 27]. In recent years, ferroptosis death were reported to be associated with antitumor immunity, which is introduced in the follow. Wang et al. reported that CD8 + T cells induce ferroptosis in tumor cells in vivo which shown the direct evidence of the connection between ferroptosis and antitumor immunity [28]. Tumor cells undergoing ferroptosis might conceivably function as arachidonic acid (AA) donors for the transcellular biosynthesis of eicosanoids, thereby participating in the generation of biologically active immunomodulatory AA metabolites that affect antitumor immunity [29]. In pancreatic cancer, the process of iron death significantly promotes disease progression and may be a potential strategy for inhibiting pancreatic cancer development. For example, GOT1 inhibition promotes pancreatic cancer cell death by ferroptosis. In this study, we identified a ferroptosis related gene signature for predicting the prognosis of pancreatic adenocarcinoma patients.

Due to the insidious and aggressive nature of PAAD, it is difficult to detect and prevent PAAD at an early stage. At the time of consultation, approximately 80% of patients have locally advanced or metastatic cancer [30]. Although multimodal therapy has been improved, surgery remains an effective therapeutic strategy for this disease. Even in conjunction with adjuvant therapy, pancreatic surgery can improve 5-year survival by only 20% [31]. Therefore, prognostic signatures for PAAD patients are urgently needed. With the advancement in the field of bioinformatics and sequencing technology, several potential prognostic evaluation methods for PAAD patients have been developed [32–34]. However, mostly genome or transcriptome parameters are analyzed in these methods, with no consideration of biological processes. Therefore, these models do not analyze the characteristics features of PAAD. Ferroptosis is a significant biological hallmark of tumors and has been demonstrated to be of value in evaluating the prognosis of patients with PAAD [36]. In this study, ferroptosis-related genes were collected, and gene expression data from public databases such as the TCGA and GEO were used to construct PAAD molecular subtypes based on ferroptosis-related genes. Next, LASSO regression was used to further compress the 10 genes to reduce the number of genes in the risk model. We used fivefold cross-validation to construct a model and analyze the confidence interval under each lambda. Hence, we selected 5 genes at lambda = − 3.75 as target genes and further selected 3 genes (ALOX5, ALOX12, and CISD1) by multivariate Cox regression analysis, and all three genes could significantly improve the performance of distinguishing between the LRG and HRG in the training sample (P < 0.05). The final model based on the 3-gene signature was as follows: RiskScore = 0.289 × ALOX5 + (− 1.359) × ALOX12 + (− 1.053) × CISD1. We found that the constructed 3-gene signature model achieved an accurate prognostic assessment of PAAD samples relative to the other methods. We also compared with previously developed gene-based signatures for PAAD, with two prognostic risk models: 20 and 36 gene signature which result showed our model obtained a more effective result with the reasonable number of genes. The performance of our prognostic risk model was further verified by using validation set data and qPCR and IHC experiments, also with pancreatic cancer cell lines in vitro validation. The functional study shown that expression of ALOX5, ALOX12, and CISD1 can regulate the migration and invasion ability of PAAD cells. Our 3-gene signature model, with fewer genes, is more accurate, reasonable, and efficient as compared to other established models.

The signature we constructed contained three genes, namely, ALOX5, ALOX12, and CISD1, all of which are closely related to tumors genesis and development. 5-Lipoxygenase (ALOX5) is a non-heme iron-containing dioxygenase that catalyzes the peroxidation of polyunsaturated fatty acids, such as arachidonic acid [37]. ALOX5 is a key enzyme that mediates lipid peroxidation and thereby leads to cell death [38]. Available evidence shows that lipid peroxidation evokes multiple types of cell death including apoptosis, pyroptosis, and ferroptosis [39]. It has also been reported that high ALOX5 expression is significantly associated with a poor prognosis in colorectal cancer, gastric cancer, clear cell renal cell carcinoma, papillary thyroid carcinoma, and other tumors [40–43]. ALOX12 gene encodes the enzyme arachidonate 12-lipoxygenase. This enzyme acts on various polyunsaturated fatty acid substrates to produce biologically active lipid intermediates, including eicosanoids and lipoxins. The ALOX12 protein plays an important role in inflammation and oxidation. Abnormal DNA methylation and genetic variation in ALOX12 are associated with various human diseases and pathological phenotypes, such as cardiovascular disease, diabetes, neurodegenerative disease, respiratory disease, cancer, and infection [44]. Many studies indicate the abnormal expression of ALOX12 in tumors, suggesting that ALOX12 may be a potential marker for many varieties of cancers. Studies in xenotransplantation models show that ALOX12 inactivation reduces the p53-mediated ferroptosis induced by ROS stress, and thus eliminates the p53-dependent tumor growth inhibition [45]. Compared with pancreatic cancer precursors and normal pancreatic ducts, the expression of ALOX12 in pancreatic cancers is significantly down-regulated and inhibits the proliferation of PAAD cells [46]. CISD1 (mitoNEET) belongs to a newly discovered class of iron-thionine (2Fe–2S). It is essential for regulating iron and ROS homeostasis in cells and plays a key role in promoting cancer cell proliferation and supporting tumor growth and metastasis [47]. Additionally, CISD1 is overexpressed in both lung adenocarcinoma and breast cancer [48, 49].

In this study, we present evidence that ALOX5, ALOX12, and CISD1 may have prognostic value in PAAD. Specifically, our in vitro results suggest that ALOX5 may be an oncogene, while ALOX12 and CISD1 may be tumor suppressor genes in PAAD. The results of our study indicate that the established 3-gene signature model could be an effective prognostic tool for patients with PAAD. However, the limitations associated with this study emphasize the need for additional analysis before the clinical application of this signature. The samples used in our study were obtained retrospectively and the study solely focused on the prognostic value and clinical significance of ferroptosis. To validate our findings for clinical applications, we need to include prospective samples and evaluate the prognostic values of other biological processes characteristic to the development of cancer.

## Conclusion

In conclusion, we propose a 3-gene signature (ALOX5, ALOX12, and CISD1) predictive model based on ferroptosis-related genes in PAAD. Despite the many drawbacks of the current analysis, this model may serve as an interesting molecular diagnostic tool to assess the prognosis and possible risk factors of PAAD.

## Supplementary Information

**Additional file 1. ** Different expressed genes (DEGs) between three clusters.

**Additional file 2. ** KEGG pathway analysis and GO functional enrichment analysis.

## Data Availability

The results shown here are in whole or part based upon data generated by TCGA (https://www.cancer.gov/tcga), KEGG (https://www.genome.jp/kegg), GO (http://geneontology.org), GEO (https://www.ncbi.nlm.nih.gov/geo).

## Abbreviations

PAAD: Pancreatic adenocarcinoma
TCGA: The Cancer Genome Atlas
GEO: Gene Expression Omnibus
NMF: Non-negative matrix factorization
ROS: Reactive oxygen species
DEGs: Differentially expressed genes
FDR: False discovery rate
KEGG: Kyoto Encyclopedia of Genes and Genomes
GO: Gene ontology
ssGSEA: Single-sample gene set enrichment analysis
GSVA: Gene Set Variation Analysis
KM: Kaplan–Meier
IHC: Immunohistochemistry
CCK-8: Cell Counting Kit-8
LRG: Low-risk groups
HRG: High-risk groups
DCA: Decision curve analysis
CCLE: Cancer Cell Line Encyclopedia
RCD: Regulated cell death
